# Grouping of tooth surfaces by susceptibility to caries: a study in 5–16 year-old children

**DOI:** 10.1186/1472-6831-4-2

**Published:** 2004-10-28

**Authors:** Paul A Batchelor, Aubrey Sheiham

**Affiliations:** 1 Department of Epidemiology and Public Health, University College London, 1-19 Torrington Place, London WC1E 6BT, UK

## Abstract

**Background:**

The decline in caries has slowed and this may be indicative of variation in the susceptibility of differing teeth to caries. This study tests the hypothesis that in children, there are groups of tooth sites that exhibit differences in caries susceptibility.

**Methods:**

Probit analysis of caries data collected from a 4-year longitudinal study of 20,000 schoolchildren aged between 5 and 16 years in 10 differing locations in the United States.

**Results:**

The development of dental caries within the mouth followed a fixed hierarchy indicating that tooth surfaces show variation in caries susceptibility. Certain teeth and tooth sites have similar susceptibilities and can be grouped, the sizes of the groups vary. The most susceptible group consists of six tooth surfaces: the buccal pits and occlusal fissured surfaces of the first molar teeth. The second group consisted of 12 sites on the second molar and premolar teeth. The group formed by the least susceptible sites included the largest number of tooth surfaces and consists of the majority of the lower anterior teeth and canines.

**Conclusion:**

Variation in the caries susceptibility of tooth surfaces exists. Surfaces can be grouped according to caries susceptibility. An effect that reduces the cariogenic challenge of one of the sites within a group is likely to affect all the other sites within the particular group.

## Background

The decline in caries that has occurred in industrialized countries over the past 30 years has been accompanied by major changes in the pattern of caries within the mouth. While the absolute levels of disease have declined, a relatively higher proportion of pit and fissured surfaces and lower proportion of approximal and smooth surfaces are involved. An additional feature in the pattern of dental caries is the existence of a surface hierarchy in susceptibility to caries [1-4]. These authors have reported that the most susceptible surfaces are pit and fissured followed by approximal surfaces on posterior teeth, and the least susceptible, approximal surfaces on anterior teeth.

There is also a reported degree of symmetry both between the upper and lower jaws in the posterior sextants [5] and left and right side of the risk of caries. The concept is so well accepted that some survey systems for recording dental caries will only examine one side of the mouth and then double the score to give the total DMFS [6]. The fact that caries occurs bilaterally in the same type of tooth suggests that when a decline or increase in caries occurs, it presents in increments of 2.

For a given DMF-T or S, there is a specific pattern of caries within a population. A working rule is that 'As caries in the population declines, caries in the least susceptible surfaces (approximal and smooth surfaces) decreases considerably more than in the most susceptible surfaces (pits and fissures)'. This pattern is independent of the presence of fluoride [7].

Furthermore, changes in mean DMF scores are not linear, but 'stepped' [4]. This stepped model of the changing patterns of caries suggests certain groups of teeth and tooth sites may have a similar 'resistance' to caries. When the resistance of one site in a group is increased, for example by fluoride, then all sites with similar resistance levels will also benefit and not become carious. Indeed, the existence of groups by resistance may explain the rapid stepped rates of decline of caries in the past 20 years [8]. A major reason for this rapid decline can be explained by the fact that groups of teeth or tooth sites with similar propensity to decay respond as a group to increase of resistance or reduction in the challenge. This would lead to a marked decline in overall caries levels rather than a gradual reduction. The present study tests the hypothesis that in children, there are groups of tooth sites by caries susceptibility.

## Methods

### Study population

This study used the data from the National Preventive Dentistry Demonstration Programme (NPDDP) in the United States [9]. The NPDDP data set was used as it contains the most extensive and comprehensive longitudinal data available on caries preventive regimes using standardised DMF criteria that have been shown to be reliable through extensive critical analyses of the project. Perhaps most importantly for this project, the caries data range was very wide: both within the individual ethnic groups and according to water fluoridation status. The NPDDP included 20,052 children aged from 5 to 16 years of age from 10 locations in the USA: five fluoridated and 5 non-fluoridated communities. The mean DMF-S at the commencement of the programme was 2.43, and was 4.51 four years later. Preventive interventions were introduced that allowed an analysis of the changes in caries patterns with the decline in caries to be examined [9-11]. The caries surface data were selected for each child at the beginning of the study.

### Statistical methods

To test the hypothesis probit analysis was used. Probit analysis is a method for examining any dose-response relationship where the dependent variable, i.e. caries, is dichotomous (caries/no caries). Since all tooth surfaces may not 'respond' in a similar manner, the problem must be formulated in terms of the proportion responding (diagnosed as caries) at each level of challenge. With probit analysis, any changes are constant in proportion so that changes on a log scale will also be constant. In a probit transformation each of the observed proportions were translated into the value of the standard normal curve below which the observed proportion of the area was found. For example, if half the subjects in a caries trial had one particular site carious, the probit value would be 0, since half the area in a standard normal distribution falls below a *z *score of 0.

When using standard normal values, negative scores can occur. To overcome this, the constant 5 was added. For example, if a particular surface scored 1 and half the subjects studied had caries, the probit value would be 0, since half of the area in a standard normal curve falls below a *z *score of 0. When the constant is added, the transformed value for the proportion becomes 5. If the observed proportion of individuals in whom the site was carious was 0.84, the probit value would be 0.34, i.e. a *z *score of 1, which would give a transformed value of 6. The actual proportion of each of the tooth sites recorded carious at each DMF-S level was calculated and replaced with the value of the standard normal curve below which the observed proportion of the area below the curve was found.

The logarithmic transformation of the data provides a linear relationship between the probability of an event occurring for any given value. The proportion of each surface within a population diagnosed as carious at a particular DMF-S score can be calculated. For example, take the occlusal surface of the first right lower molar. At a DMF of 1, a certain proportion of this site within a population would be carious. This proportion would increase for a DMF of 2, and so on.

For each DMF-S score, the percentage of tooth surfaces exhibiting caries was calculated to give a probability score of between 0 and 1. Subsequently, the log transformation of the probabilities for each DMF-S against the actual DMF-S score was plotted for every surface. The common reference value, of 0.5 outlined above was used to establish the susceptibility of each surface to a given caries challenge. As some random variation can be expected, the susceptibilities are grouped within bands rather than as individual sites.

To establish whether a hierarchy for caries susceptibility exists, the probability of finding each site carious for a given DMF-S score was calculated. The probability was calculated by adopting a common reference value for the proportion of the tooth sites that become carious. In the present study 0.5 was used to provide the most accurate value, although any value of proportion can be used. The question can then be phrased in terms of the value of the DMF-S at which 50% of the sites or teeth would be expected to have become carious. The probability scores derived are then aggregated to produce an overall picture of tooth and site susceptibilities.

The probability of an event occurring ranges from zero to 1. As the overall DMF score rises, the probabilities of any site being carious will change. For example, if a group of 128 individuals, each having a DMF-S score of 1 with all sites exhibiting the same propensity for caries. The distribution of sites affected within the mouth should be random, the probability for a particular site being carious would then be 1/128. If, however, the group all had a DMF-S of 128, the relative probability of finding a particular site carious remains the same, but this time the absolute probability changes from 1/128 to 1.

As the DMF score increases the probabilities alter. However, once a probability of 1 has been reached no further increase is possible. Thus, when examining changes in the distribution of caries at different DMF-S scores, the ratio between individual probability scores is unimportant. The crucial factor is the overall ranking exhibited by the probabilities for each site. The order of susceptibly will be determined by the relative values of the probabilities. Whether an individual site is twice as likely to become carious as another cannot be determined using this approach. However, certain sites may exhibit similar probabilities. For example, a particular site on the left hand side of the mouth may have a similar mean probability as the corresponding site on the right hand side. Other factors may influence the distribution of probabilities. For example, it has been suggested that fluoride has a more beneficial effect on approximal sites when compared to occlusal.

The probabilities derived are for each site for each individual and are then aggregated for the sample population. The aggregation of probabilities gives rise to a distribution, approximately normal in character, and the mean of this distribution is subsequently reported and used in the analyses.

Data analyses were performed using SPSS. The data files of the NPDDP were supplied by the Rand Corporation in ASCII format and subsequently read onto the mainframe system. Two of the five data files were utilised in this project: the master file containing the demographic information of each individual and the clinical file containing the status of each tooth site.

## Results

**Figure 1:**
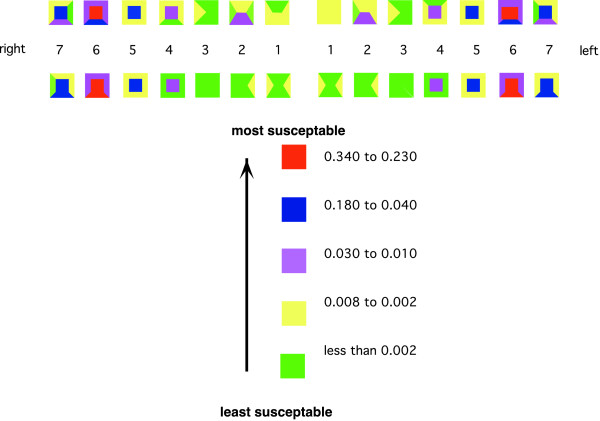
Distribution of probabilities of site susceptibilities.

Figure 1 shows the distribution of probabilities of caries grouped into 5 categories. The categories were formed by grouping together sites with similar probabilities of having experienced caries. The most susceptible groups of sites were defined as having a probability of being carious within the range 0.34 to 0.23, the next group 0.18 to 0.04, then 0.03 to 0.01, then 0.008 to 0.002 and, finally, the least susceptible sites which formed the remaining group (Figure 1).

The emerging pattern indicates a left:right side symmetry with the propensity of attack similar for the two sides of the mouth. Furthermore, there is a degree of symmetry between the upper and lower jaws in the posterior sextants. For the anterior sextants, teeth in the upper jaw are more prone to attack than those in the lower jaw.

An important finding is the relative sizes of the groups by the probability of susceptible sites having experienced caries. The group with most susceptible sites consisted of six tooth surfaces: the pit and fissured surfaces of the first molar teeth. The second group consists of 12 sites on the second molar and premolar teeth. The fifth group, and least susceptible group, is the largest and consists of the majority of the lower anterior teeth and canines.

The groups, in order of susceptibility, allowing for some left:right asymmetry, were:

1. occlusal surfaces of 1st molars and buccal pits of lower 1st molars;

2. occlusal surfaces of 2nd molars and buccal surfaces of lower 2nd molars and occlusal surfaces of all 2nd premolars;

3. occlusal surfaces of 1st premolars, palatal surfaces of upper lateral incisors, approximal surfaces of 1st molars, lingual surfaces of lower 1st molars and buccal surfaces of upper 1st molars and palatal surfaces of upper 2nd molars;

4. all approximal surfaces of 2nd premolars, all approximal surfaces of upper 1st premolars, mesial and lingual surfaces of lower 2nd molars and distal and buccal surfaces of upper 2nd molars, approximal surfaces of upper central incisors, some approximal surfaces of upper and lower lateral incisors, all approximal surfaces of lower central incisors and distal approximals of upper canines, and approximal surfaces of 2nd molars; and

5. all surfaces of lower canines, buccal and mesial and labial aspects of upper canines, all smooth and approximal surfaces of lower 1st premolars, smooth surfaces of lower central incisors, approximal surfaces of lateral incisors (Figure 1).

**Figure 2:**
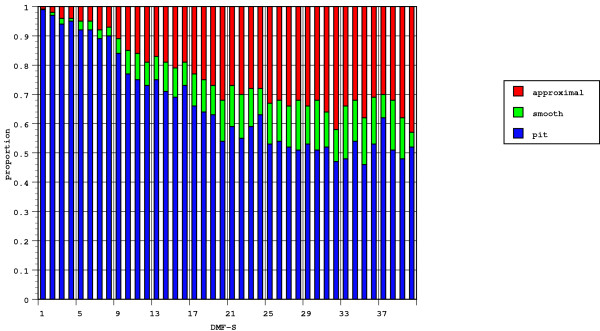
Proportion of each tooth surface type affected by caries at each DMF-S score.

The next analysis was to establish the probability of finding a particular surface type carious for each DMF score. The probability, by surface, was converted into a ratio as in Figure 1. Figure 2 combines the findings shown in Figure 1 with the patterns by tooth surface. To facilitate comparison by surface, the probability of each surface type was calculated, the total for each DMF-S score being equal to one. As the DMF score increased, the ratio of smooth to approximal to pit and fissured surfaces changes, although for the lower DMF scores, any major change in the ratios did not occur until a DMF-S of 9. Only then did the contribution of lesions on approximal or smooth surfaces make a significant contribution to the overall DMF-S.

## Discussion

This study shows that that a number of tooth sites exhibit similar susceptibilities to caries. Susceptibility of tooth sites is not only similar for homologous pairs but also for grouped sites. For example, at a DMF score of 1, six sites have very similar probabilities of being carious. These involve the pit and fissured surfaces of all first molar teeth. Most authors, whilst accepting a degree of left:right symmetry in caries attack, have assumed that it is either the same site affected on the left hand side of the mouth as on the right [12-16].

This study reported that precise symmetry of caries did not occur by site but rather that symmetry of caries existed by groups of sites. For example, in the anterior incisor region, where several sites have similar propensities, the left:right symmetry may have been lost because the mirror image site had not undergone cavitation. However, due to the similarities in susceptibilities, another site on the opposite or even the same side has cavitated. In both cases symmetry will be lost. What exists is groups of teeth by susceptibility to caries.

The concept of groups of susceptible sites has a number of important implications. If the application of a caries preventive strategy leads to a reduction in either the attack intensity or an increase in the resistance of the sites within a group to a value at which a particular site was protected then all sites in the group would also be protected. Depending upon the size of the group, several sites may well be protected. The existence of groups of sites goes some way to explain the stepped nature exhibited by changing patterns of caries. This may explain the apparent rapidity with which DMF levels decreased initially with the decline in caries reported in many industrialised countries since the 1970s. The adoption of a preventive strategy such as the use of fluoride toothpaste to reduce the attack intensity or increase in resistance for the least susceptible group, would lead to substantial savings in the total number of cavitated sites.

There is a change in the changing ratio of smooth and approximal sites on the one hand, and pit and fissured surfaces on the other, for different levels of DMF-S, confirming findings by Burt [17], Dummer et al., [18], and Vehkalahti et al., [19]. For example, at a DMF-S of 1, the ratio of pit and fissured to smooth or approximal surfaces is 99:1. At a DMF-S of 10, the ratio had changed to 3:1. However, the changing ratios should be considered against the overall decline in caries.

When considering the hierarchy of susceptibility by groups of sites the types of sites must be considered. With a change in the surface type ratio in which pit and fissured surfaces predominate to one in which approximal surface involvement becomes important, the consequences of the latter are smaller. The sites with a similar high propensity for caries attack at a DMF of 10, which includes a higher proportion of approximal surfaces, are not necessarily ten times as great as for an individual with a DMF of 1. This has important implications for planning preventive strategies. More effort would be required to reduce caries from low to very low levels than from high to low caries.

Finally, the mathematical relationships identified by Batchelor and Sheiham [20] describing the distribution of caries at the population level could be combined with the findings of this study, to develop a model to assess the impact of caries preventive strategies. The model would help provide a scientific basis on which to formulate caries preventive strategies.

## Conclusions

The findings show that there is a hierarchy by tooth types and sites in the pattern of dental caries attack in children. While it was not possible to identify precisely the order that each tooth or site succumbed to caries, groups of sites or teeth can be placed in a hierarchy of risk of caries. This concept expands the proposals of a hierarchy of 'within mouth' zones [21-23]. More importantly, there are groupings of tooth sites by susceptibility to caries. The size of the groupings varies. The impact of preventive agents that increased the resistance or reduced the intensity of the challenge affects most sites within a particular group. The larger sized groups occur at high caries levels. Increasing their resistance or lowering the intensity of the caries challenge would lead to a substantial drop in cavitated sites providing the agent or agents offered sufficient protection for any single site in the larger groups.

## Competing interests

The author(s) declare that they have no competing interests.

## Authors' contributions

PAB conceived, undertook the design of the study, performed the statistical analysis and drafted the manuscript. AS participated in the study design and coordination and helped to draft the manuscript. Both authors read and approved the final manuscript.

## Pre-publication history

The pre-publication history for this paper can be accessed here:


